# RISC in PD: the impact of microRNAs in Parkinson's disease cellular and molecular pathogenesis

**DOI:** 10.3389/fnmol.2013.00040

**Published:** 2013-11-20

**Authors:** Sabrina M. Heman-Ackah, Martina Hallegger, Mahendra S. Rao, Matthew J. A. Wood

**Affiliations:** ^1^Department of Physiology, Anatomy and Genetics, University of OxfordOxford, UK; ^2^Center for Regenerative Medicine, US National Institutes of HealthBethesda, MD, USA

**Keywords:** Parkinson's disease, microRNA (miRNA), ubiquitin-proteasome system, autophagy, apoptosis, mitochondria, dopaminergic neurons, iPS cells

## Abstract

Parkinson's disease (PD) is a debilitating neurodegenerative disease characterized primarily by the selective death of dopaminergic (DA) neurons in the substantia nigra pars compacta of the midbrain. Although several genetic forms of PD have been identified, the precise molecular mechanisms underlying DA neuron loss in PD remain elusive. In recent years, microRNAs (miRNAs) have been recognized as potent post-transcriptional regulators of gene expression with fundamental roles in numerous biological processes. Although their role in PD pathogenesis is still a very active area of investigation, several seminal studies have contributed significantly to our understanding of the roles these small non-coding RNAs play in the disease process. Among these are studies which have demonstrated specific miRNAs that target and down-regulate the expression of PD-related genes as well as those demonstrating a reciprocal relationship in which PD-related genes act to regulate miRNA processing machinery. Concurrently, a wealth of knowledge has become available regarding the molecular mechanisms that unify the underlying etiology of genetic and sporadic PD pathogenesis, including dysregulated protein quality control by the ubiquitin-proteasome system and autophagy pathway, activation of programmed cell death, mitochondrial damage and aberrant DA neurodevelopment and maintenance. Following a discussion of the interactions between PD-related genes and miRNAs, this review highlights those studies which have elucidated the roles of these pathways in PD pathogenesis. We highlight the potential of miRNAs to serve a critical regulatory role in the implicated disease pathways, given their capacity to modulate the expression of entire families of related genes. Although few studies have directly linked miRNA regulation of these pathways to PD, a strong foundation for investigation has been laid and this area holds promise to reveal novel therapeutic targets for PD.

## Introduction

### Parkinson's disease

PD is a progressive neurodegenerative disease which manifests as a debilitating movement disorder with late cognitive sequelae. Pathologically, PD is characterized by the selective loss of DA neurons in the midbrain substantia nigra pars compacta with four clinical hallmarks resulting from the loss of dopamine signaling in the striatum. These four hallmarks, which clinically define Parkinsonism, are pill-rolling tremor, cogwheel rigidity, bradykinesia/akinesia and postural instability (Savitt et al., 2006). With progression of the disease, the neurodegenerative process spreads to involve other brain regions. Most notably, the forebrain is commonly affected in late PD leading to cognitive decline and dementia (Jellinger, 2012). Despite pharmacologic therapy with dopamine replacement (Cotzias, 1968; Antonini and Cilia, 2009), and recent advances in surgical treatment with deep brain stimulation (Pereira and Aziz, 2006; Pereira et al., 2007; Farris and Giroux, 2011), there remains a void in understanding and inhibiting the underlying progressive neurodegeneration that defines PD. It has thus become a significant research focus of recent years to examine the molecular mediators underlying this process with the objective of translating this understanding to the development of new therapeutic approaches.

In recent years, researchers have identified genetic mutations which cause approximately 10% of PD cases (Klein and Westenberger, 2012). Investigations into the precise link between genetic mutation and disease, and the etiology of the 90% of sporadic PD cases, have led to the identification of molecular pathways that culminate in initial DA neuron injury and the resulting DA neuron death and progressive neurodegeneration. The primary pathways which have been implicated as mediators of the degenerative process are the protein quality control pathways, the ubiquitin-proteasome system (UPS) and autophagy pathway, as well as apoptosis, mitochondrial quality control, and DA differentiation and maintenance; the proposed role of each of these pathways in PD pathogenesis is described in detail below. Defects in these pathways may explain the underlying etiology of both genetic and sporadic PD pathogenesis. Thus, great interest has developed in understanding mechanisms of endogenous regulation of these pathways. Toward this end, the characterization of miRNA function in PD pathogenesis has become of particular interest and the potential of these molecules to serve as pathway modifiers for therapeutic intervention in PD is increasingly appreciated.

### miRNA biogenesis and function

**Figure 1:**
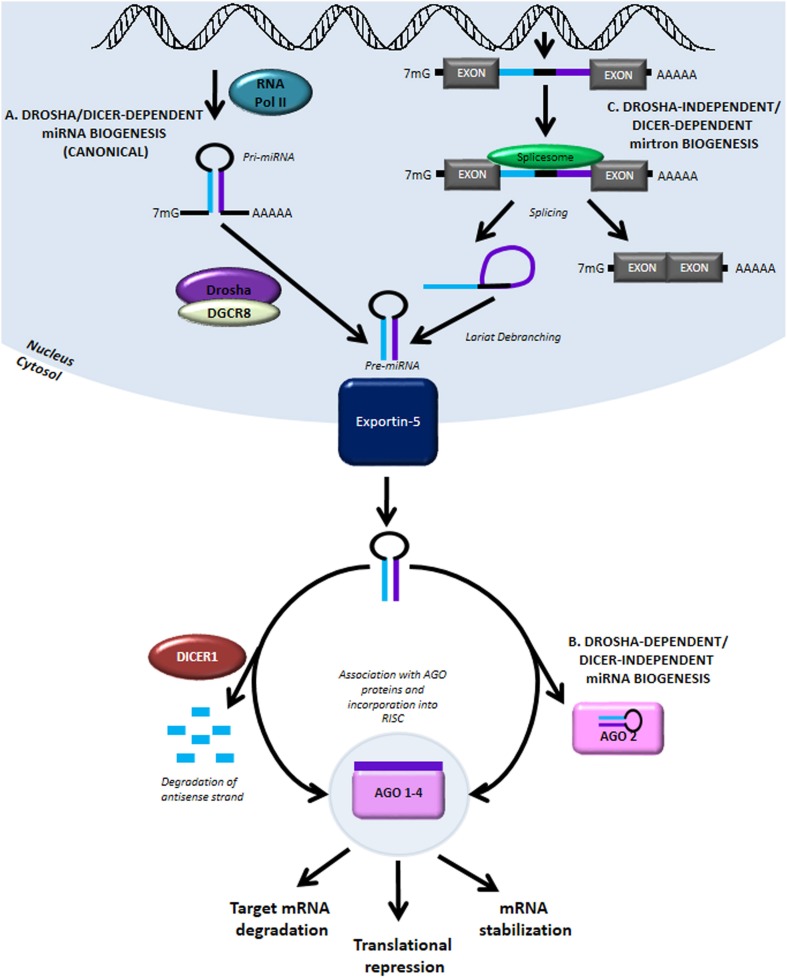
**MiRNA biogenesis and function. (A)** The canonical miRNA biogenesis pathway is Drosha- and Dicer-dependent. It begins with RNA Pol II-mediated transcription of genomic loci containing miRNA genes. The primary transcript is referred to as pri-miRNA and like other RNA Pol II transcripts contains a 5′ 7-methylgunaosine cap and 3′ poly-A tail. The pri-miRNA is processed in the nucleus by Drosha and DGCR8 to form pre-miRNA. The pre-miRNA is then exported into the cytoplasm by Exportin-5 where it is further processed by the RNase III enzyme, Dicer. Interaction with Dicer leads to hairpin cleavage and degradation of the anti-sense strand of the miRNA duplex, while the mature guide strand is complexed with members of the Argonaute family of proteins to form a functional RNA-induced silencing complex (RISC). **(B)** In a Drosha-dependent and Dicer-independent non-canonical pathway of miRNA biogenesis, the cytoplasmic pri-miRNA undergoes processing by AGO2, although the precise mechanism of miRNA maturation via this pathway remains unclear. **(C)** Another non-canonical pathway involving Drosha-independent/Dicer-dependent biogenesis generates mirtrons, transcribed from intronic sequences and obtained by splicing and lariat-debranching. Mature miRNA can act via three primary methods: (1) destabilization and cleavage of target mRNA, (2) translational repression, and (3) mRNA stabilization.

miRNAs are endogenous regulators of gene expression. These small non-coding RNAs (ncRNAs) can be transcribed by RNA polymerase II (RNA Pol II) from two primary genomic loci: miRNA genes and intronic sequences. In the canonical biogenesis pathway (Figure 1A), transcription from miRNA genes yields pri-miRNAs which are processed in the nucleus by the Drosha/DGCR8 microprocessor complex to produce pre-miRNAs. The processed pre-miRNAs are then exported to the cytoplasm by Exportin-5, where they are further cleaved by the RNase III enzyme Dicer to produce a mature miRNA duplex. The mature guide strand is 20–22 nucleotides in length and associates with Argonaute proteins, AGO 1–4, to form a functional RNA-induced silencing complex (RISC). The anti-sense strand, denoted by miRNA*, was previously thought to be degraded; recent evidence suggests that some of these may have biological activity. The mature miRNA is then responsible for aligning the RISC to target mRNA by binding at complementary seed sequences in the 3′UTR. This association of target mRNA with the miRNA-containing RISC most commonly results in down-regulation of gene expression by translational repression and recruitment of protein complexes causing deadenylation and degradation of target mRNA. Conversely, miRNAs have also been shown to stabilize transcripts under certain cellular conditions (Melton et al., 2010).

The canonical pathway of miRNA biogenesis is also referred to as Drosha-dependent/Dicer-dependent miRNA biogenesis. In the much rarer, non-canonical Drosha-dependent/Dicer-independent miRNA biogenesis pathway, the exported pre-miRNA is processed by AGO2 in the cytoplasm to produce the mature guide strand (Figure 1B). miRNAs generated by another non-canonical pathway, termed Drosha-independent/Dicer-dependent miRNA biogenesis, are referred to as mirtrons (Figure 1C). In this pathway, mirtrons are spliced from the intronic sequences of transcribed genes, forming a lariat structure which is de-branched to form the pre-miRNA hairpin structure. After export from the nucleus, mirtrons function similarly to miRNA generated via the canonical pathway. Further discussion of miRNA biogenesis pathways can be found in detailed reviews on this topic (Bartel, 2009; Ameres and Zamore, 2013).

Our understanding of the role ncRNAs play in development and disease has expanded rapidly over the last decade. Recent insights are providing evidence that other classes of ncRNAs may contribute to transcriptome alterations in PD. For a more detailed discussion on the roles of long ncRNAs and small vault RNAs in PD, the reader is referred to the following references (Minones-Moyano et al., 2013; Wu et al., 2013). miRNAs in particular have been identified as critical regulators of gene expression in a number of normal biological and pathophysiological processes. miRNA dysregulation has been convincingly linked to a number of neurodegenerative diseases. For a discussion of the role miRNAs play in Alzheimer's disease and Huntington's disease, the reader is referred to the enclosed references (Delay et al., 2012; Sinha et al., 2012). Recently discovered relationships between PD-related genes and miRNAs and consideration of miRNA regulation of cellular and molecular pathways that have been implicated in PD pathogenesis are explored further in this review.

### miRNA-based therapeutics

A better understanding of the role miRNAs play in the cellular and molecular pathogenesis of PD will undoubtedly contribute to the development of novel miRNA-based therapies. It is therefore, imperative to make a number of important considerations in moving miRNAs from molecular targets to viable therapeutics. First, because of their ability to modulate the expression of families of related genes that participate in common cellular and molecular pathways, miRNAs hold great potential to restore balance to dysregulated pathways at the onset and very early stages of PD (Ouellet et al., 2006). However, the ability to apply a miRNA-based therapy within this critical time period is currently limited by an inability to accurately diagnose PD at these early stages (Akhtar and Stern, 2012). Thus, the detection of biomarkers, development of sensitive imaging techniques, and discovery of clinical criteria that can discern PD in these early stages will be critical for the development of miRNA-based therapeutics with disease-altering potential (Akhtar and Stern, 2012). Additionally, given the mechanism of action of RNAi-based therapies, it is important to consider off-target effects resulting from transcriptional and translational repression of unintended miRNA targets (Rao et al., 2009). In the development of miRNA-based therapies for PD, it is also necessary to consider the route of delivery to achieve therapeutic levels of the miRNA in the brain (Boudreau et al., 2011). Although targeted delivery of small RNAs to the brain has presented a formidable challenge in the past, recent evidence suggests that exosomes may be used to deliver exogenous cargo, including nucleic acids, to the brain, providing a novel means of overcoming this current limitation to the translation of miRNA-based therapies (Alvarez-Erviti et al., 2011). Finally, an important limitation to the testing of miRNA therapeutics is the lack of an animal model that recapitulates key features of PD pathology (Beal, 2010). Although animal models have been developed that display nigostriatal degeneration, an animal model that demonstrates Lewy body pathology, a key feature of human Parkinsonism, remains to be discovered. This underscores a potential difference between the cellular and molecular mechanisms of Parkinson's symptomatology in existing models compared to that in humans, presenting a challenge for the accurate detection and application of miRNAs that would modulate these pathways for therapeutic benefit. Despite these limitations, it has been demonstrated that an integrated analysis of PD mouse models, existing human cell culture models and novel human iPSC-derived DA neuron models can provide an accurate prediction of clinical efficacy for drug treatments of PD, indicating promise for utilizing such an integrated approach for testing miRNA-based therapies. Specifically, it has been shown that a subset of compounds which were found to have pharmacologic benefit in mouse models of PD had a protective effect in MPP+-treated SH-SY5Y cells, and further that a subset of those were protective in MPP+-treated iPSC-derived TH+ neurons (Peng et al., 2013). Importantly, those compounds which were effective in both SH-SY5Y and iPSC-derived TH+ neurons had the greatest benefit when translated to patient care (Peng et al., 2013). Similar integrative approaches using non-human primate and human iPSC culture models have been successful in predicting miRNA-based therapeutic efficacy (Chan and Kocerha, 2012). Thus, this integrative approach provides a means of thoroughly testing miRNA-based therapeutics with a readout that can provide an indication of clinical effectiveness as never previously attainable.

## Interaction between miRNA and PD-related genes

### PD-related genes

**Figure 2:**
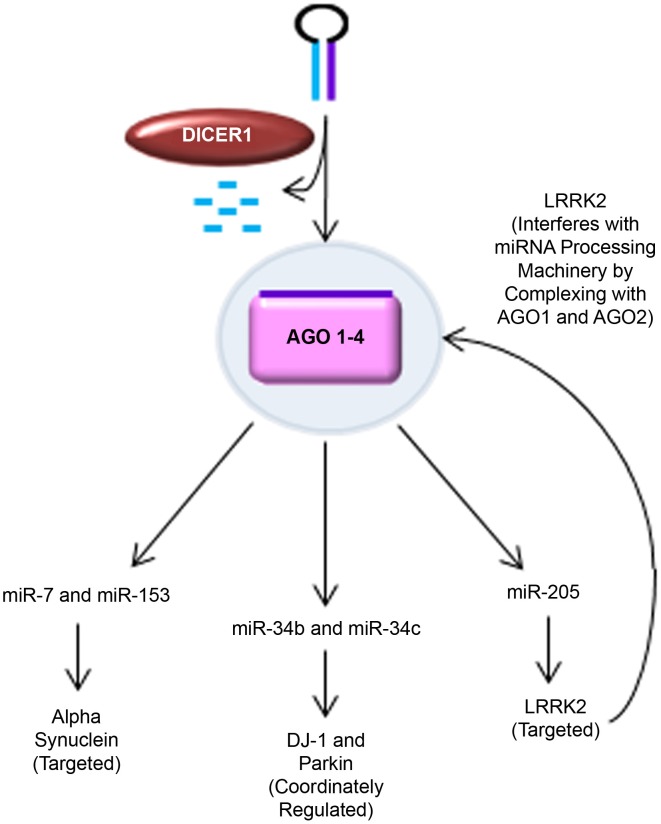
**MiRNA regulation of PD-related genes**. Novel findings supporting the role of miRNA in regulating key PD-related genes are shown. Alpha synuclein was predicted to have seed sequences in the 3′ UTR for miR-7 and miR-153. This was experimentally validated by Junn et al. (miR-7) and Doxakis (miR-7 and miR-153) in several PD-relevant models, including mice challenged with MPTP and MPP+-treated SH-SY5Y cells. DJ-1 and Parkin were found to be coordinately regulated with miR-34b and miR-34c expression in PD patient brains and SH-SY5Y cells (Minones-Moyano et al., 2011). LRRK2 was putatively predicted to be regulated by miR-205 and experimentally confirmed in post-mortem PD patient brain samples, primary cultured mouse cortical neurons, and LRRK2 R1441G BAC transgenic mice (Cho et al., 2013). Interestingly, pathogenic LRRK2 has also been found to interfere with miRNA processing machinery by complexing with drosophila AGO1 and human AGO2 (Gehrke et al., 2010).

Of the 28 distinct chromosomal loci that have been convincingly linked to PD, only six have been demonstrated to cause heritable monogenic PD (Klein and Westenberger, 2012). Table 1 lists these six genes, their mode of inheritance, and the cellular pathways currently thought to be affected. The following sections discuss studies which have identified miRNA interactions with these genes; these are summarized in Figure 2. No studies to date have identified direct miRNA interactions with PINK1 or ATP13A2.

**Table 1:** **PD-related genes**.

**PD-related gene symbol**	**Gene name**	**Mode of inheritance**	**Relevance in PD**
PARK1/PARK4	Alpha synuclein	Autosomal dominant	Point mutations and gene multiplications cause synucleinopathy
PARK2	Parkin	Autosomal recessive	Loss of E3 ubiquitin-ligase activity leads to aberrancies of ubiquitin-proteasome system and mitophagy
PARK6	PINK1	Autosomal recessive	Dysfunction of mitochondrial quality control
PARK7	DJ-1	Autosomal recessive	Dysfunction of mitochondrial quality control
PARK8	LRRK2	Autosomal dominant	Gain of kinase activity; proposed aberrancies of membrane trafficking and cytoskeletal dynamics
PARK9	ATP13A2	Autosomal recessive	Causes Kufor-Rakeb syndrome (atypical PD); normally located in lysosomal membrane, retained in ER in disease

Mutations in these six genes have been demonstrated to cause inherited monogenic forms of PD. Several miRNAs have been identified to regulate particular genes in this list, including alpha synuclein and LRRK2. A miRNA-gene interaction has been described for DJ-1 and Parkin, however the mechanisms remain elusive. Each of these interactions is described in detail in the text. Of note, no miRNA have yet been described to interact with PINK1 or ATP13A2.

### Alpha synuclein levels are regulated by miR-7 and miR-153

Alpha synuclein has a long-known role in PD pathogenesis. Point mutations, duplications, and triplications in this gene are sufficient to cause the death of DA neurons via alpha synuclein aggregation and neurotoxicity (Singleton et al., 2003). Additionally, alpha synuclein is a major component of Lewy bodies, a pathologic hallmark of PD (Spillantini et al., 1997, 1998). A dose-response relationship of this gene has been described, in which individuals with alpha synuclein multiplications develop PD at an earlier onset age and with increasing severity associated with dementia (Singleton et al., 2003; Farrer et al., 2004). Where the overproduction of a gene product is the mechanism by which the gene contributes to PD pathogenesis, there is a clear implication that miRNA-mediated gene suppression might hold potential to improve the disease phenotype.

Using this as a basis for investigation, two groups independently discovered miR-7 as a regulator of alpha synuclein expression (Junn et al., 2009; Doxakis, 2010). Junn et al. demonstrated that miR-7 levels are higher in the substantia nigra and striatum of mice, compared to cerebral cortex and cerebellum. MiR-7 levels were found to be 40 times higher in neurons than in astrocytes, and as well alpha synuclein was detected in neurons but not astrocytes. This provides support for endogenous miR-7 regulation of alpha synuclein levels in neurons. To further understand the potential implications of miR-7 in PD, the authors investigated miR-7 levels in MPP+ treated SH-SY5Y cells, and MPTP-intoxicated mice, finding elevated alpha synuclein levels, and reduced miR-7 levels in both cases. This indicates that a reduction in miR-7 may contribute to nigrostriatal degeneration. Doxakis et al. independently described a similar result of alpha synuclein regulation by miR-7, as well as miR-153. Additionally, overexpression of miR-7 and miR-153 in cultured cortical neurons caused a 30–40% reduction in endogenous alpha synuclein levels. Taken together these results suggest that delivery of miR-7 and miR-153 may represent an appealing therapeutic strategy to promote neuroprotection in patients with known alpha synuclein gene multiplications.

### LRRK2 is targeted by miR-205

The precise normal function of the leucine-rich repeat kinase 2 (LRRK2) has yet to be determined, although recent evidence suggests involvement in membrane trafficking (West et al., 2005; Biskup et al., 2006, 2007; Gloeckner et al., 2006; Hatano et al., 2007; Sakaguchi-Nakashima et al., 2007; Alegre-Abarrategui and Wade-Martins, 2009; Alegre-Abarrategui et al., 2009; Lee et al., 2010a,b,c; Tong et al., 2010; Vitte et al., 2010) and cytoskeletal dynamics (Jaleel et al., 2007; Gandhi et al., 2008; Gillardon, 2009; Parisiadou et al., 2009; Lin et al., 2010). Mutations in LRRK2 have been identified as the most common cause of dominantly inherited PD (Brice, 2005; Lesage et al., 2006; Ozelius et al., 2006; Healy et al., 2008) and importantly, variation in the LRRK2 gene has been implicated as a risk factor for sporadic PD (Kett and Dauer, 2012). LRRK2 is thought to contribute to PD pathogenesis through a gain-of-function mechanism (Kett and Dauer, 2012). Indeed, the most common LRRK2 mutation, a glycine to serine substitution at position 2019 (G2019S), leads to increased activity in the activation loop of the kinase domain (West et al., 2005; Greggio et al., 2006; Jaleel et al., 2007). The finding that LRRK2 inhibition blocks neurotoxicity *in vitro* and *in vivo* provides additional support for a gain-of-function mechanism (Greggio et al., 2006; Smith et al., 2006; Lee et al., 2010a).

Congruent with the notion that increased LRRK2 activity contributes to PD pathogenesis, Cho et al. demonstrated that normal LRRK2 levels are higher in the frontal cortex of sporadic PD and PD with dementia (PDD) patients compared to non-pathological controls (NPC) (Cho et al., 2013). LRRK2 mRNA levels were found to be comparable between these groups, suggesting post-transcriptional regulation, as would be mediated by miRNA. MiR-205 was identified as a putative regulator of LRRK2 by target prediction algorithms. Further investigation revealed significantly lower levels of miR-205 in the frontal cortex and striatum of PD and PDD patients, compared to NPC. In primary mouse cortical neurons, inhibition of miR-205 caused upregulation of LRRK2 protein expression whereas overexpression of miR-205 repressed LRRK2 protein expression. Strikingly, wild type mouse midbrain DA neurons displayed a high level of miR-205 and in transgenic mice overexpressing R1441G (arginine to glycine at position 1441) mutant LRRK2, miR-205 treatment rescued impairment of neurite outgrowth. This study described a novel regulatory role for miR-205 with LRRK2. Given that over-activity of LRRK2 is suggested to cause PD, therapeutic replacement of miR-205 is an attractive target for therapeutic intervention, particularly for sporadic cases in which LRRK2 levels were found to be elevated and miR-205 levels were found to be low.

It is worth noting that new insights are beginning to reveal a reciprocal role for LRRK2 in regulating miRNA biogenesis by interfering with the miRNA processing machinery through complexing with drosophila AGO1 and human AGO2 (Gehrke et al., 2010). Further investigation will be required before a precise role for LRRK2 regulation of miRNA biogenesis and translational repression can be fully appreciated.

### DJ1 and parkin are coordinately regulated with miR-34b and miR-34c

Miñones-Moyano et al. profiled the miRNA expression pattern in post-mortem tissue from PD patient brains, discovering a dysregulation of miR-34b and miR-34c (Minones-Moyano et al., 2011). The authors identified miR-34b and miR-34c downregulation at advanced stages of PD finding that miR-34 reduction compromises neuronal viability by mitochondrial dysfunction and production of reactive oxygen species in an SH-SY5Y neuroblastoma culture model. They further characterized that the miR-34b/c reduction is correlated with decreased expression of DJ1 and Parkin, noting that these proteins were indeed downregulated in PD brain tissue as well. This provides evidence that miR-34b/c downregulation may involve DJ1 and Parkin, however the precise mechanism by which this interaction occurs remains unclear.

It is an important consideration that as DA neurons degenerate throughout the life of PD patients, post-mortem tissue samples often lack the quantity and quality of DA neurons necessary to delineate whether the molecular defects observed are truly relevant to the DA neurons that produce pathology, or whether they are simply a result of the lack of the diseased neurons in the region investigated. To date, examinations of post-mortem brain tissue have provided the most physiologically relevant means of investigating the molecular mechanisms of PD. Newer models such as PD patient-specific induced pluripotent stem cell (iPSC)-derived DA neurons will aid in further interpreting and probing miRNA mediated defects in human DA neurons and delineating the precise molecular events which lead to disease.

### Predicted miRNA targeting PD-related genes

Although significant progress has been made in our understanding of the role miRNA play in regulating PD-related genes, much remains to be answered. A small number of miRNA have been identified to regulate the six monogenic PD-causing genes, whereas many more which can be identified as putative targets by multiple target prediction algorithms (Enright et al., 2003; John et al., 2004; Lewis et al., 2005; Grimson et al., 2007; Betel et al., 2008, 2010; Friedman et al., 2009; Maragkakis et al., 2009a,b; Garcia et al., 2011), have not been experimentally validated (Table 2). Although central to discovery of miRNA targets, this underscores both the limitations of target prediction algorithms as well as the necessity for models which more closely recapitulate the pathophysiology of PD to delineate the specific effects of these miRNAs in the context of PD.

**Table 2:** **Putative miRNA targeting PD-related genes**.

**PD-Related gene**	**TargetScan**	**miRanda**		**DIANA microT**
Alpha synuclein (PARK1/PARK4)	**miR-7**	miR-539	miR-431	**miR-7**
	miR-7a	**miR-7**	miR-23b	**miR-153**
	miR-7b	miR-488	miR-23a	
	**miR-153**	miR-504	miR-425	
	miR-223	miR-487b	miR-216a	
	miR-214	miR-374b	miR-340	
	miR-761	miR-374a	miR-133b	
	miR-3619-5p	miR-144	miR-133a	
		miR-495	miR-125a-3p	
		**miR-153**	miR-485-5p	
		miR-129-5p	miR-17	
		miR-599	miR-106b	
		miR-223	miR-106a	
		miR-449b	miR-519d	
		miR-449a	miR-20b	
		miR-34-c-5p	miR-20a	
		miR-34a	miR-93	
		miR-148a	miR-342-3p	
		miR-152	miR-454	
		miR-148b	miR-130a	
		miR-539	miR-494	
		miR-361-5p	miR-222	
		miR-182	miR-221	
		miR-29b		
Parkin (PARK2)	***miR-181a***	miR-379	miR-488	miR-147
	***miR-181b***	miR-544	miR-216a	***miR-181c***
	***miR-181c***	miR-488	miR-203	***miR-181a***
	***miR-181d***	miR-590-3p	miR-187	***miR-181d***
	miR-4262	miR-708	miR-185	***miR-181b***
		miR-28-5p	miR-505	
		miR-599	miR-320d	
		miR-363	miR-320c	
		miR-367	miR-320b	
		miR-25	miR-320a	
		miR-92b	miR-140-5p	
		miR-92a	miR-876-5p	
		miR-32	miR-222	
		miR-125a-3p	miR-221	
		miR-146b-5p	miR-199b-5p	
		miR-146a	miR-199a-5p	
		miR-155	miR-200a	
		miR-758	miR-141	
		***miR-181c***	miR-19b	
		***miR-181b***	miR-19a	
		***miR-181a***	miR-200b	
		***miR-181d***	miR-200c	
		*miR-543*	miR-429	
PINK1 (PARK6)	miR-532-3p	miR-346		^*^
		miR-124		
		miR-506		
		miR-216b		
		miR-340		
		miR-217		
DJ-1 (PARK7)	^*^	miR-128		^*^
		miR-758		
		miR-539		
		miR-216b		
		miR-544		
		miR-365		
		miR-874		
LRRK2 (PARK8)	**miR-205**	miR-384	miR-103	***miR-181c***
	miR-205a	miR-590-3p	miR-708	***miR-181a***
	miR-205b	miR-185	miR-28-5p	***miR-410***
	***miR-19a***	**miR-205**	miR-30a	***miR-19b***
	***miR-19b***	miR-543	miR-30b	***miR-19a***
	***miR-181a***	miR-410	miR-30c	***miR-454***
	***miR-181b***	miR-136	miR-30d	**miR-205**
	***miR-181c***	miR-301b	miR-30e	***miR-181b***
	***miR-181d***	miR-301a	miR-328	***miR-181d***
	miR-4262	***miR-454***	miR-186	
	miR-130a	***miR-19b***	miR-429	
	miR-130c	***miR-19a***	miR-200c	
	miR-301a	miR-382	miR-200b	
	miR-301b	miR-144	miR-23b	
	miR-301b-3p	miR-384	miR-23a	
	***miR-454***	miR-376c	miR-340	
	miR-721	***miR-181c***	miR-129-5p	
	miR-4295	***miR-181d***	miR-32	
	miR-3666	***miR-181b***	miR-363	
		***miR-181a***	miR-367	
		miR-381	miR-92b	
		miR-300	miR-92a	
		miR-141	miR-25	
		miR-200a	miR-9	
		miR-107		
ATP13A2 (PARK9)	^*^	miR-199a-5p	miR-424	
		miR-199b-5p	miR-15a	
		miR-24	miR-15b	
		miR-299-3p	miR-497	
		miR-122	miR-433	
		miR-16	miR-873	
		miR-195		

This table presents miRNA which are independently predicted to target PD-related genes by three different open-access target prediction algorithms. Bolded miRNA have been experimentally validated, whereas bolded and italicized miRNAs were identified by all three algorithms, but have not been experimentally confirmed.

* , No targets found using the following parameters: miRNA mapped to “Conserved sites for miRNA families broadly conserved among vertebrates” or those listed as “Conserved” in TargetScan; score threshold of 7.3 in DIANA-microT 3.0.

## Global miRNA dysregulation in PD pathogenesis

Many compelling lines of evidence suggest a role for global miRNA dysregulation from interference with miRNA processing machinery in neurodegeneration. Defects due to Dicer knockout are evident as early as the embryonic stage in mice, resulting in defects in cell proliferation (Murchison et al., 2005) and differentiation (Kanellopoulou et al., 2005). Importantly, mouse Dicer knockouts demonstrate lethality before neurulation (Bernstein et al., 2003). Depletion of Dicer in the developing mouse neocortex leads to reduction in cortex thickness and defective cortical layering, and the mouse dies shortly after weaning (De Pietri Tonelli et al., 2008). Several studies have demonstrated the appearance of hallmarks of neurodegeneration in mouse brains with conditional knockout of Dicer in cortical, hippocampal, cerebellar, motor and striatal neurons, as well as astroglia (Schaefer et al., 2007; Cuellar et al., 2008; Davis et al., 2008; Kawase-Koga et al., 2009; Haramati et al., 2010; Tao et al., 2011). Furthermore, loss of the DGCR8 component of the microprocessor complex has been shown to produce neuronal and behavioral defects in mice (Stark et al., 2008). Additionally, mice with lineage specific defects in miRNA processing machinery have been shown to develop defects in spinal motor neurons, reminiscent of spinal muscular atrophy (Haramati et al., 2010) as well as defects in forebrain neurons, consistent with Alzheimer's pathology (Hebert et al., 2010).

One of the first studies to demonstrate a role for miRNAs in the maintenance of midbrain DA neurons was conducted by Kim et al. (2007). Cre-mediated deletion of Dicer in ES cells at stage 4 of differentiation, when post-mitotic DA neurons first arise, resulted in complete loss of DA neuron accumulation at stage 5, while the generation of other mature neuronal classes was less affected (i.e., GABAergic neurons and TUJ1 positive neurons). Importantly, the phenotype was rescued by transfection of low molecular weight RNA species, indicating that the observation indeed results from the lack of mature small RNA species, including miRNA. Furthermore, in a rodent model, conditional knockout of Dicer under the control of the dopamine transporter (DAT) induced apoptosis in substantia nigra, and behavioral studies demonstrated dramatically reduced locomotion, reminiscent of the phenotype of human PD. This seminal study provided some of the first evidence that miRNAs have a unique role in the development and maintenance of midbrain DA neurons. We further discuss the roles of specific miRNA in developing DA neurons in section DA neuron differentiation and maintenance.

## Role of miRNA in pathways implicated in PD molecular pathogenesis

**Figure 3:**
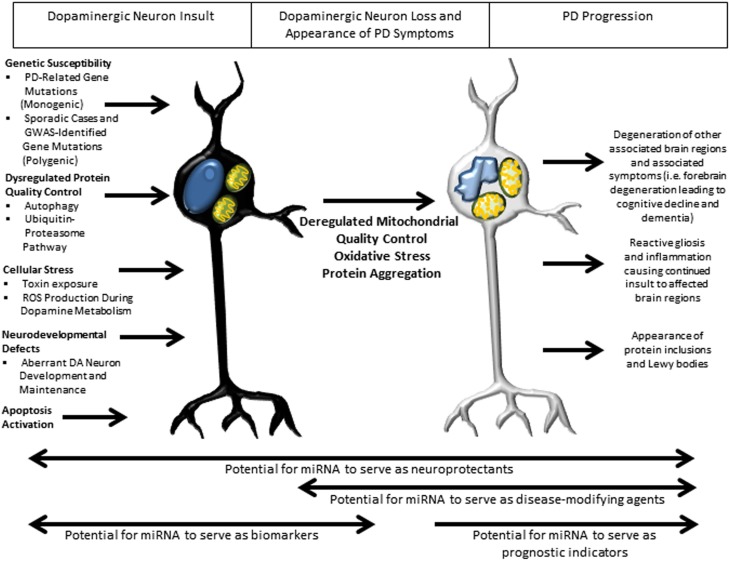
**Overview of pathways implicated in PD pathogenesis and potential roles of miRNA in therapy**. Several well-studied cellular pathways have now been implicated in PD pathogenesis, with an apparent convergence on mitochondrial damage, oxidative stress and protein aggregation leading to DA neuron death and downstream sequelae of the progressive pathogenic process. In the earliest stages of insult, miRNA may have their greatest therapeutic benefit as biomarkers, with the potential to identify patients before the appearance of symptoms, allowing early therapeutic intervention. Throughout the disease course, miRNA might serve as neuroprotectants owing to their ability to regulate families of genes. Later in the disease course there is the potential for miRNA to serve as disease-modifying agents and prognostic indicators of disease progression.

In recent years, dysfunction of a number of critical pathways has been directly implicated in the pathogenesis underlying PD (Figure 3). Such aberrant pathways are thought to be a common effector leading to PD in both inherited and sporadic cases. Whereas the PD-related gene interactions with miRNAs discovered to date generally describe one miRNA interacting with one gene, perhaps the most impactful role of miRNAs is their ability to regulate families of related genes. In this regard, understanding the roles of these pathways in PD pathogenesis and the mechanism by which miRNA may form regulatory networks with the gene families they comprise will be essential to our ability to harness the potential of miRNA to serve as neuroprotective and disease-modifying agents in PD therapy.

### Protein quality control

A well described cellular phenomenon observed in nearly all forms of neurodegeneration, including PD, is abnormal protein accumulation (Ross and Poirier, 2004). This protein accumulation is primarily thought to be a result of dysfunctional protein clearance, although it is worth noting that it may also be the consequence of protein overproduction, as is seen in patients with alpha synuclein gene multiplications (Singleton et al., 2003). The two primary mechanisms by which cells perform clearance of protein aggregates are the UPS and autophagy (Cook et al., 2012). Whereas the UPS is the primary mechanism by which damaged or misfolded proteins are degraded by the cell, it has limited capacity to handle protein aggregates, such as those that form the characteristic Lewy bodies of PD. In contrast, the autophagy pathway has the ability to rid the cell of large protein aggregates as well as aged and damaged organelles, including mitochondria (mitophagy). Since damaged mitochondria and protein aggregation are suggested events upon which PD pathogenesis converges, autophagy has recently been recognized as a pathway which may unify many divergent cellular etiologies of PD pathogenesis.

#### Ubiquitin-proteasome system

The UPS is comprised of E1 ubiquitin-activating enzymes which generate a reactive thiol ester between the E1 cysteine residues and the C-terminal glycine of ubiquitin, E2 ubiquitin-conjugating enzymes which carry ubiquitin to the protein substrate, and E3 ubiquitin ligase enzymes which catalyze the ligation of ubiquitin to the protein substrate (Hershko and Ciechanover, 1992). Whereas E1 and E2 enzymes have non-specific activity, E3 ubiquitin ligases confer target specification. After the addition of a minimum of four ubiquitin molecules, the protein substrate is carried into the 20S proteolytic core of the 26S proteasome for cleavage (Hershko and Ciechanover, 1998; Ciechanover et al., 2000; Ciechanover and Brundin, 2003).

Dysfunction of the UPS has been implicated in both genetic and sporadic forms of PD. In brains from sporadic PD patients, ubiquitinated proteins and components of the UPS appear in Lewy bodies (Lennox et al., 1989; Lowe et al., 1990; Li et al., 1997; Auluck et al., 2002; McNaught et al., 2002; Schlossmacher et al., 2002). Furthermore, Parkin has been identified as an E3 ubiquitin ligase, mutations in which have been confirmed to cause an autosomal recessively inherited form of early-onset PD (Klein and Westenberger, 2012). Interestingly, patients with these mutations tend to display a loss of DA neurons, but no Lewy body accumulation, indicating that Parkin activity may be required for LB formation (Cook et al., 2012). It is worth noting that the controversial (Maraganore et al., 2004; Healy et al., 2006) ubiquitin carboxy-terminal hydrolase L1 (UCHL1/PARK5), gene acts in the UPS to hydrolyze the E1-ubiquitin bond formed by E1 ubiquitin-activating enzymes. Thus, despite uncertainty as to whether UCHL1 is a true PD susceptibility factor, its link to PD and the UPS overall provides evidence supporting a role for UPS dysfunction in PD.

The UPS has also been recently identified as a regulator of miRNA biogenesis (Smibert et al., 2013). In drosophila, interference with the UPS leads to accumulation of AGO1. Additionally, the stability of AGO2 in mouse cells is linked to miRNA availability. AGO2 is decreased with Dicer-knockout, causing a loss of miRNAs, but rescued by proteasome blockade. Together these findings implicate the UPS as an essential regulator of miRNA biogenesis by controlling levels of miRNA processing enzymes and RISC components. Although this study provides initial evidence for an interaction between the UPS and miRNA function, no studies as of yet have directly linked miRNA dysregulation of the UPS to PD.

#### Autophagy

Autophagy is the only known pathway by which cells can degrade organelles and protein aggregates that cannot be processed by the proteasome (Lynch-Day et al., 2012). There are three main types of autophagy, which are active to varying degrees in different cell types: chaperone-mediated autophagy (CMA), microautophagy, and macroautophagy. CMA is a process wherein misfolded proteins are carried to lysosomes by chaperone proteins and transferred across the lysosomal membrane for degradation by hydrolases. In microautophagy, the lysosome invaginates to take in misfolded proteins, protein aggregates and other cytosolic substrates. Once inside the lysosome, the vesicle contents are quickly hydrolyzed. In macroautophagy large sections of the cytosol are engulfed by a double-membrane vesicle known as an autophagosome. The autophagosome then fuses with lysosomes, releasing lysosomal enzymes and hydrolases into its lumen to degrade its contents.

Compared to other cell types, neurons have a higher basal rate of autophagy, necessary due to the inability to dilute aged and damaged particles via mitosis (Son et al., 2012). However, aberrancies in this process have long been postulated as a source of neuronal death and neurodegeneration (Son et al., 2012). Macroautophagy is the most clearly linked to PD pathogenesis thus far (Cook et al., 2012). Several studies have described involvement of the aforementioned PD-related genes in the autophagy pathway, including LRRK2 and alpha synuclein (Bandyopadhyay and Cuervo, 2007; Alegre-Abarrategui and Wade-Martins, 2009). Cells harboring alpha synuclein mutations and gene multiplications have been found to demonstrate greater autophagic clearance of normal mitochondria. In cell culture models pre-aggregated alpha synuclein is resistant to degradation and impairs autophagy. Mechanistically these aggregates impair overall macroautophagy by reducing autophagosome clearance. This potentially could contribute to the increased cell death observed in aggregate-bearing cells (Tanik et al., 2013). Furthermore, Parkin and PINK1 have been described to act in concert to remove damaged mitochondria by promoting their degradation via mitophagy (Lee et al., 2010b; Matsuda et al., 2010; Narendra et al., 2010). It is suggested that both reduced and overactive autophagy are detrimental to the health of DA neurons, thus making miRNAs particularly compelling as therapeutic targets given their key properties of containing endogenous regulatory mechanisms and functioning to fine-tune the activity of gene families.

**Figure 4:**
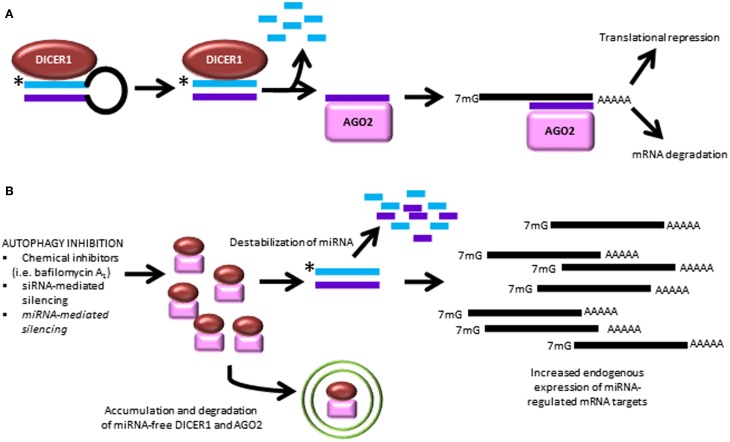
**Autophagy inhibition causes dysregulated miRNA processing and function. (A)** The canonical pathway of miRNA biogenesis is shown leading to the two best characterized actions of miRNA to cause degradation of mRNA targets and interfere with mRNA translation. **(B)** Recent findings demonstrate that autophagy inhibition leads to break-down of miRNA-free Dicer and AGO2, causing downstream miRNA instability and overexpression of untargeted transcripts (Gibbings et al., 2012, 2013). ^*^, antisense miRNA strand, which is degraded during processing.

***Regulation of miRNA homeostasis by autophagy.*** A recent paper by Gibbings et al. demonstrated a novel role for the autophagic pathway in miRNA homeostasis (Gibbings et al., 2012, 2013). In this study, it was found that autophagy regulates miRNA biogenesis and function via selective degradation of DICER1 and AGO2. These enzymes accumulate in cells lacking the key autophagy genes, ATG5, ATG6, ATG7, as well as the selective autophagy receptor, CALCOCO2. Interestingly, when the selective receptor CALCOCO2 is depleted, ubiquitinated AGO2 accumulates, suggesting a role for ubiquitin-mediated clearance, in addition to ubiquitin-independent recognition events. Importantly, pre-miRNA, miRNA and miRNA^*^ strands are not degraded by autophagy. However, when autophagy is inhibited, the ability of AGO2 to bind miRNA duplexes is decreased, and this prevention of miRNA loading onto AGO2 leads to miRNA instability and decay. Ultimately, this release of translation repression leads to overexpression of proteins which are regulated by miRNA (Figure 4). This is the first study to demonstrate a role for autophagy in regulating miRNA homeostasis.

***miRNA downregulation of autophagy in PD.*** In another recent paper, Alvarez-Erviti et al. expanded on their previous findings that LAMP-2A and hsc70, two autophagy mediators, are low in PD brains by investigating the modulation of the CMA pathway by miRNA (Alvarez-Erviti et al., 2013). Target prediction identified four putative LAMP-2A targeting miRNAs and two putative hsc70 targeting miRNAs, which had also been previously reported to be increased in PD brains (Kim et al., 2007). MiR-106a caused a dose-dependent decrease in hsc70 3′UTR activity, as did miR-224 for LAMP-2A. Ultimately LAMP-2A was found to be regulated by four and hsc70 by three of the identified miRNAs. Strikingly, alpha synuclein levels increased in response to all of the miRNAs tested, presumably because of decreased activity of the CMA pathway. Importantly, three miRNA targeting LAMP-2A and three targeting hsc70 were significantly increased in PD brain substantia nigra pars compacta and amygdala, associated with a decrease in protein levels. Thus, this paper provides additional evidence supporting an intricate interplay between autophagy and miRNA pathways, in this case providing evidence that miRNA may be useful modulators of CMA-related dysfunction in PD.

Although we are only beginning to uncover the roles of miRNA in regulating autophagy in the context of PD, already, there are critical insights gained from studies in other systems that foreshadow investigation in PD-relevant cellular and animal models. Since it has been suggested that cancer and neurodegeneration are manifestations of opposing cellular dysfunctions (Plun-Favreau et al., 2010), studies of miRNA regulation of autophagy in cancer may provide an invaluable foundation upon which investigations of miRNA regulation of autophagy in neurodegeneration may build (Frankel and Lund, 2012).

### Apoptosis

Many of the mutations and toxins associated with PD are linked with mitochondrial function, providing a connection between known risk factors and cellular physiology that could explain PD pathophysiology. DA neurons in particular are sensitive to mitochondrial stressors and toxins. The maintenance of ion gradients underlying neuronal excitability signifies one of the major energetic burdens for neurons. The energy is generated in mitochondria, the sites of cellular respiration, which also leads to the production of damaging superoxide and other reactive oxygen species (Surmeier et al., 2012).

Superoxide and reactive oxygen species are only a subset of the stressors that cells encounter and that damage macromolecules and organelles. In response they can either attempt to regain cellular homeostasis or, if the severity or duration of the encountered stress is too harmful, they can induce cell death. For example, when cellular clearing processes like autophagy or mitophagy fail, apoptosis can be induced. This mechanism normally guarantees the removal of cells that are not able to adapt to the encountered stress or regain cellular homeostasis. However, programmed cell death can have devastating consequences—in many neurodegenerative diseases, like PD, these dying cells will not be replaced.

Apoptosis is a critical pathway of programmed cell death in which cells undergo a well-defined series of steps that result in the ultimate fragmentation of the nucleus and blebbing of the cell membrane. A period of massive apoptosis is a necessary step in the normal development of the human brain to define functional synaptic connectivity. Just as critical however, is that this process ends at the formation of post-mitotic neurons, as these neurons must then survive throughout the life of the organism. In recent years, significant progress has been made in understanding the role of this pathway in neuronal development and differentiation, as well as the molecular mediators regulating these processes, thus paving the way for future investigations of molecular aberrations that may lead to disease. Particularly, iPSCs originating from PD patients have made it possible to study the molecular events leading to apoptosis in DA neurons. Several studies have demonstrated that DA neurons generated from iPSCs with familial mutations in LRRK2 and alpha synuclein exhibited greater sensitivity to oxidative stress and had a higher number of apoptotic neurons (Byers et al., 2011; Nguyen et al., 2011; Reinhardt et al., 2013). This increased vulnerability to oxidative stress was also observed in iPSC-derived DA neurons from idiopathic PD patients (Sanchez-Danes et al., 2012). Apoptotic markers were also detected in post-mortem brains of PD patients (Tatton et al., 1998; Hartmann et al., 2000; Mogi et al., 2000; Tatton, 2000) as well as in animal models of PD, in particular models generated by the DA neurotoxin MPTP (Tatton and Kish, 1997; Viswanath et al., 2001).

In the following section we describe how miRNAs modulate programmed cell death during development and disease. MiRNAs would be well positioned to help neurons regain homeostasis upon cellular stresses. MiRNAs can act as restorers of homeostasis by resuming normal gene expression through negative feedback loop mechanisms. When miRNAs are components of a positive feedback loop, they can induce new gene expression patterns that help overcome cellular stress. MiRNAs themselves can also become the targets of regulation and their loss can affect downstream gene expression.

#### Apoptosis-promoting miRNA are associated with neuronal differentiation

Aranha et al. identified three miRNAs with previously described pro-apoptotic functions to be intimately involved with the differentiation of CNS subtypes from neural stem cells, including neurogenesis and gliogenesis (Aranha et al., 2011). This study was based on recent evidence implicating pro-apoptotic molecules, such as p53, caspases and Bcl-2 in differentiation and development, and aimed to further understand the role of pro-apoptotic mediators in neural differentiation processes. The authors characterized miR-14, let7a, and miR-34a upregulation during neural stem cell differentiation, and demonstrated that their expression, particularly miR-34a, coincided with the appearance of post-mitotic immature neurons. The use of apoptosis promoting miRNAs during neuronal development is consistent with the high rate of neuronal apoptosis that occurs throughout early development during the process of pruning, a normal developmental program that facilitates the formation of efficient synaptic configurations. Interestingly, however, the increased expression of these miRNAs was not associated with increased cell death. The authors hypothesize that the role in this context may be more to control cell cycle exit and mitotic inhibition, given the timing of expression and appearance of neuronal and glial subtypes. This study provides crucial evidence for a novel role of pro-apoptotic miRNAs in neural differentiation.

Conversely, in a Drosophila model of aging it was shown that miR-34 is up-regulated in aging brains and deletion of miR-34 leads to accelerated brain ageing, neural degeneration, defective protein folding, and a decline in survival. Rescue with miR-34 was sufficient to mitigate mutant effects, wherein inclusion formation was slowed, protein retained greater solubility and neural degeneration was suppressed. Furthermore, overexpression of miR-34 had a neuroprotective effect in a transgenic Drosophila model with genetic background that lead to overexpression of neurotoxic poly-glutamines (Liu et al., 2012).

#### miR-29b restricts apoptosis and promotes neuronal maturation

In 2011, Kole et al. became the first to identify a mammalian miRNA with the ability to inhibit the BH3-only family of apoptosis initiators in neurons (Kole et al., 2011). Importantly, the focus of this paper was on post-mitotic neurons, consistent with the biological condition in which it would be most important to restrict further death of neurons. MiR-29b was found to be selectively enriched in sympathetic ganglion, cerebellar and cortical neurons isolated from postnatal day 28 (P28) mice, compared to the lower expression level in postnatal day 5 (P5) neurons. MiR-29b overexpression was sufficient to inhibit apoptosis in response to three independent stimuli: nerve growth factor (NGF) deprivation, endoplasmic reticulum (ER) stress and DNA damage. NGF deprivation leads to c-Jun phosphorylation, BH3-only induction and subsequent cytochrome c release, caspase activation and cell death. It was determined that miR-29b acts downstream of c-Jun phosphorylation, but upstream of cytochrome c release, prompting the investigation of a possible interaction with BH3-only proteins. Strikingly, miR-29b was found to interact with five out of eight members of the BH3-only family of proteins. The BH3-only protein family repression was responsible for blocking apoptosis only in mature neurons, and protein expression failed to be induced on NGF deprivation in P28 neurons, while being induced in P5 neurons. The findings of this paper are particularly important because it is remarkable example of a single miRNA interacting with multiple members of the same family of proteins. These proteins have been well-characterized to serve redundant functions. Thus, it would be essential that a miRNA capable of affecting the apoptotic pathway through interactions with this family regulate many of its members. MicroRNA-29 has been previously reported to have roles in Alzheimer's disease (Hebert et al., 2008), as well as cancer (Pekarsky et al., 2006; Mott et al., 2007; Wang et al., 2008; Gebeshuber et al., 2009; Park et al., 2009; Han et al., 2010), highlighting the cell- and context-specific nature of its role in regulating apoptosis. Overall, this paper indicates miR-29b as an important miRNA with the ability to fine-tune apoptosis activation and regulation via key members of the BH3-only proteins. These findings certainly warrant further investigation in the context of PD.

#### Downregulation of miRNA through UPR leads to activation of apoptosis

Extensive research has made a link between accumulation of unfolded proteins and apoptosis in PD. One of the pathways activated by accumulating unfolded proteins in the ER is the unfolded protein response (UPR). UPR signaling requires IRE1α, an ER membrane localized endonuclease that upon induction of UPR cleaves the mRNA of XBP1 and this cleavage leads to a change of its open reading frame and activation of XBP1's transcription factor activity. If ER stress is too lasting, it can trigger cell death. This is mediated through the protease caspase-2 as an early apoptotic switch. Upton et al. now report that IRE1α is the ER stress sensor that activates caspase-2 and does so through a mechanism involving miRNAs (Upton et al., 2012). Upon UPR activation, the RNase activity of IRE1α cleaves selected microRNAs (miR-17, -34a, -96, -125b) that normally repress translation of caspase-2 mRNA, consequently increasing caspase-2 abundance and activating apoptosis. Whether these events occur in neurodegenerative disease with UPR activation remains to be investigated.

### Mitochondrial miRNA

How mitochondrial dysfunction affects miRNAs and how mitochondrial epistasis is regulated by miRNAs is under much investigation. Some evidence came from an *in vitro* study showing that RISC loading with small duplex RNA is inefficient in the absence of ATP, which is generated by mitochondria (Yoda et al., 2010). In addition, disruption of mitochondrial ATP production in human cell lines leads to decreased RISC activity caused by failing RISC assembly (Huang et al., 2011). Therefore mitochondrial dysfunction in PD could lead to an overall weakening of miRNA pathways. Localization of the RISC complex has been shown in multivesicular bodies, like late endosomes, P-bodies, and stress granules. More recently, miRNAs have been detected in mitochondria, providing a link between mitochondrial function and miRNA regulation.

MiRNAs specifically enriched in mitochondria have received much attention. Their localization allows regulation of mitochondrial transcription and translation, but potentially could indicate that miRNAs are stored in mitochondria. In these studies miRNAs were isolated from mitochondria from a variety of tissues and cell lines including rat liver (Kren et al., 2009), mouse liver (Bian et al., 2010), a human epithelial carcinoma cell line (HeLa) (Bandiera et al., 2011), a human embryonic kidney cell line (HEK293) (Sripada et al., 2012) and human skeletal muscle cells (Barrey et al., 2011). Technically, it was crucial that the isolated mitochondria were highly purified and treated with RNase to remove mitochondria-bound cytosolic RNA prior to miRNA extraction to avoid unwanted contamination.

In 2011, Bandiera et al. provided the first comprehensive view of mitochondrial associated miRNAs in HeLa cells (Bandiera et al., 2011). After confirming the presence and activity of AGO2 localized to the mitochondria, the authors set out to identify mitochondrial associated miRNAs, which they termed mitomiRs. Comparing isolated cytosolic and mitochondrial RNA fractions, the authors identified 57 differentially expressed miRNAs, 13 of which were significantly enriched in mitochondria. Interestingly, of the 13 miRNA identified, three (miR-1974, -1977 and -1978) map to the mitochondrial genome. It is important to note, however, that these three mitomiRs map specifically to tRNA and rRNA genes and were removed from miRBase, among other comprehensive microRNA databases, putting into question whether they are bona fide miRNAs or breakdown products of tRNAs and rRNAs. Among the notable findings from this investigation, the authors were able to ascertain that the mitomiRs had predicted targets on mitochondrial genes, including those essential for ATP synthesis coupled electron transport, translation initiation, cell cycle and mitochondrial translation.

A further study by Sripada et al. took a similar approach in fractionating cells to isolate mitochondrial RNAs (Sripada et al., 2012). In this report, however, the authors chose to investigate miRNA from two different commonly studied cell lines, HEK293 and HeLa, by next generation RNA sequencing on the Illumina HiSeq2000 platform, with enrichment for small RNA. The authors note that the same 13 miRNA identified by Bandiera et al. were represented in their data. The differences observed in the most abundant miRNAs highlight that in addition to technical variation in the cellular fractionation procedure, differences in the method of probing miRNA expression may produce significant variance. Importantly, RNA sequencing allowed the identification of several putative novel mitochondrial miRNAs. Interestingly, only 35 miRNA were similarly expressed in mitochondria of HEK293 and HeLa cells.

The overlap in the above data sets is surprisingly small and could be explained by mitochondrial isolation procedure and the miRNA profiling platform chosen. This again highlights the importance of consistent sub-fractionation procedures, consistent miRNA probing methods, but possibly also the tissue specificity of mitochondrial miRNA expression. Thus transcriptome analysis of PD post-mortem tissue, DA neurons from PD animal models, and patient-specific iPSC-derived neurons will provide an important link between the mitochondrial dysfunction in disease and changes in miRNA profiles. Several studies of miRNA function in DA neurons have already laid the path for future investigation.

### DA neuron differentiation and maintenance

#### miR-133b regulates Pitx3

The seminal paper for its first demonstration of the role of miRNAs in midbrain DA neuron development by Kim et al. has been mentioned previously (Kim et al., 2007). The authors set out to determine specific miRNAs that mediated the findings described above (see section Global Mirna Dysregulation in PD Pathogenesis). The authors used a qPCR panel to quantify 230 miRNA precursors on samples derived from PD and control midbrain, cerebellum and cerebral cortex, finding miR-133b to be specifically deficient in PD samples. A similar reduction in miR-133b was observed in Pitx3 mutant mice. This regulation was confirmed *in vitro* by luciferase reporters, showing that Pitx3 activates the miR-133b promoter and secondly that the Pitx3 3′UTR is negatively regulated by miR-133b overexpression.

**Figure 5:**
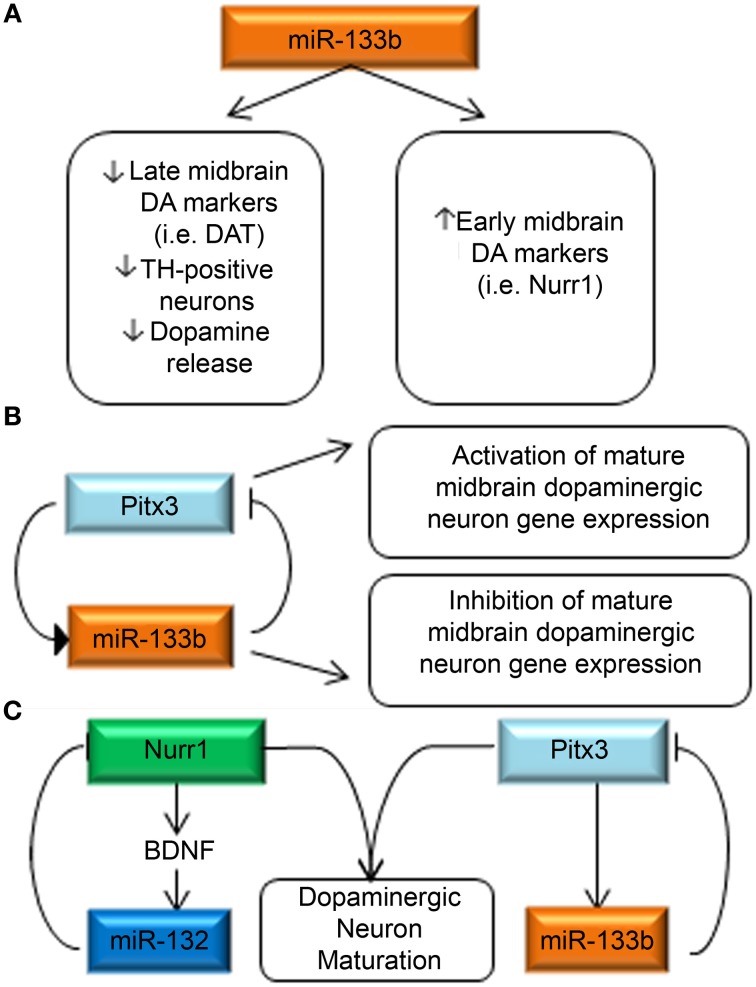
**miRNA regulation of key DA neurodevelopmental genes. (A)** miR-133b decreases the expression of late DA neuron marker signatures, while increasing the expression of early DA neuron markers (Kim et al., 2007). **(B)** Pitx3 is capable of inducing miR-133b expression, while miR-133b is reciprocally capable of regulating Pitx3 expression. The authors hypothesize a negative feedback loop in which Pitx3 concurrently activates expression of DA maturation genes and miR-133b and when miR-133b levels reach a threshold consistent with maturation, they inhibit continued expression of Pitx3. **(C)** Summary of miRNA interactions with DA neuron-relevant genes. Two primary investigations have separately identified miR-132 (Yang et al., 2012) and miR-133b (Kim et al., 2007) as regulators of Nurr1 and Pitx3, respectively. It is worth noting that SNPs in both of these genes have recently been identified as potential causes of spontaneous Parkinsonism highlighting the relevance of DA neurodevelopmental events in PD pathogenesis.

Interestingly, in primary embryonic rat midbrain cultures, overexpression of miR-133b caused decreased expression of late DA neuron markers, such as DAT, and resulted in the formation of fewer TH-positive neurons, and lower dopamine release. Inhibition of miR-133b had the opposite effect to increase the expression of late DA neuron markers. It is counterintuitive that low miR-133b expression levels are found in human Parkinsonism, as well as rodent Parkinson's models, while high expression of the miR-133b causes fewer mature DA neurons to form. However, these findings would be consistent with a role for miR-133b in a negative feedback circuit. Thus, the authors hypothesize that miR-133b normally functions to suppress Pitx3 expression post-transcriptionally, while Pitx3 induces midbrain DA gene expression and transcription of its own regulator, miR-133b (Figures 5A,B). In support of this hypothesis, the authors note their finding that overexpression of a Pitx3 transgene lacking the 3′UTR was capable of partially reversing miR-133b mediated DAT suppression. This finding is also in concert with growing evidence which suggests that the same miRNAs can function to fine-tune gene expression in a context-specific manner.

A recent description of a miR-133b null mouse model suggests that miR-133b does not play a significant role in midbrain DA neuron development and maintenance *in vivo*. The animals have normal numbers of midbrain DA neurons during development and aging with unchanged dopamine levels in the striatum. Further the suggested miR133b target, Pitx3, is unaffected as is the expression of DA genes tested (Heyer et al., 2012).

#### miR-132 regulates DA differentiation and maintenance

Another key transcription factor for midbrain DA development is also subjected to miRNA-mediated regulation and was described in 2011 by Yang et al. who profiled miRNAs in purified DA neurons (Yang et al., 2012). A mouse ES cell line expressing GFP under control of the TH promoter was generated and allowed a FACS sort for GFP positive cells after 13 days of DA differentiation. A qPCR-based array method was used to profile relative expression of miRNAs in the GFP-positive, GFP-negative and neural progenitor populations. MiR-132 was identified to be more than fivefold higher in GFP-positive cells than in neural progenitors. MiR-132 overexpression suppressed DA neuron differentiation, whereas miR-132 down-regulation promoted the differentiation of DA neurons. Bioinformatic analysis revealed Nurr1 as a putative target of miR-132, which was confirmed by luciferase reporter assay. The authors further investigated the interaction between Nurr1, BDNF and miR-132, as Nurr1 was previously known to regulate BDNF, and BDNF had been previously identified to regulate miR-132 expression. They found that Nurr1 and miR-132 expression levels were inversely correlated, while BDNF and miR-132 were coordinately expressed. They hypothesize that a homeostatic mechanism thus exists between Nurr1, BDNF and miR-132 (Figure 5C).

In addition to its role in DA differentiation through regulation of BDNF and Nurr1, miR-132 has been found to have a pro-survival effect which may contribute to DA neuron maintenance. A delicate balance between acetylcholine and dopamine signaling exists within the striatum (Threlfell and Cragg, 2011). It has been shown that overexpression of acetylcholinesterase (AChE), the enzyme that catabolizes acetylcholine, has a pro-apoptotic effect (Park et al., 2004). In PD, declining dopamine signaling in the striatum may contribute to an acetylcholine imbalance resulting in relative overexpression of AChE, further promoting the death of DA neurons (Llinas and Greenfield, 1987). miR-132 has been found to inhibit AChE, thus suggesting a neuroprotective role for this miRNA in DA neurons (Shaked et al., 2009). This finding remains to be investigated in human neuronal models.

Taken together these studies have identified miRNAs as significant regulators of genes necessary for the differentiation and maintenance of DA neurons. The contrasting results seen in the aforementioned studies of miR-133b highlight the importance of further investigation of these particular miRNAs and their gene-specific interactions in the context of PD patient-specific iPSC-derived DA neurons.

## Conclusion

This review has presented recent advances in the emerging field which aims to elucidate the role of miRNAs in PD. Many of these studies which have arisen in just the last decade have provided strong evidence that dysregulated miRNA serves as an essential molecular trigger which potentiates pathogenesis in PD. Here we have highlighted studies which demonstrated a role for global miRNA dysregulation in aberrant development and maintenance of DA neurons, a finding which complements the growing notion that neurodegeneration may be a late manifestation of neurodevelopmental disease. Further, we have included a discussion of miRNAs that have been found to regulate the protein clearing pathways, the UPS and autophagy, as well as those involved in apoptosis, mitochondrial maintenance, and DA neuron differentiation, aberrancies in each of which have been previously implicated as cellular and molecular mediators of PD. Despite the dedicated work of many, and the rapidity with which advances have occurred, there remain several unanswered questions about the molecular pathogenesis of PD. The development of novel and robust technologies, such as patient-specific iPSC-derived DA neurons, will further the study of PD on cellular and molecular levels as never previously attainable. By applying evidence obtained from pathway-specific analyses with these new tools, researchers are closing the gap between our knowledge of the disease process and our desire to advance its treatment.

### Conflict of interest statement

The authors declare that the research was conducted in the absence of any commercial or financial relationships that could be construed as a potential conflict of interest.
